# 2-Carb­oxy-1-(3-nitro­phen­yl)ethanaminium perchlorate

**DOI:** 10.1107/S1600536809051319

**Published:** 2009-12-12

**Authors:** Wen-Xian Liang, Yan Li, Gang Wang, Zhi-Rong Qu

**Affiliations:** a Ordered Matter Science Research Center, College of Chemistry and Chemical, Engineering, Southeast University, Nanjing 210096, People’s Republic of China

## Abstract

In the cation of the title compound, C_9_H_11_N_2_O_4_^+^·ClO_4_^−^, the conformation is stabilized by an intra­molecular N—H⋯O hydrogen bond. In the crystal packing, centrosymmetrically related cations inter­act through inter­molecular O—H⋯O hydrogen bonds involving the carb­oxy groups, forming dimers. The dimers and the perchlorate anions are further linked into layers parallel to the *ab* plane by C—H⋯O and N—H⋯O hydrogen-bonding inter­actions.

## Related literature

For the synthesis of β-amino acids, see: Cohen *et al.* (2002 ▶); Qu *et al.* (2004 ▶); Zhao (2007 ▶). For hydrogen-bond motifs, see: Bernstein *et al.* (1995 ▶); Etter *et al.* (1990 ▶).
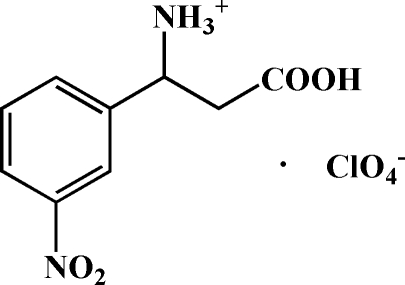


## Experimental

### 

#### Crystal data

C_9_H_11_N_2_O_4_^+^·ClO_4_^−^
*M* *_r_* = 310.65
Triclinic, 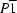

*a* = 7.5932 (8) Å
*b* = 7.8843 (1) Å
*c* = 11.8615 (6) Å
α = 94.745 (3)°
β = 99.780 (7)°
γ = 116.323 (4)°
*V* = 617.11 (7) Å^3^
*Z* = 2
Mo *K*α radiation
μ = 0.35 mm^−1^
*T* = 293 K
0.45 × 0.30 × 0.15 mm

#### Data collection

Rigaku SCXmini diffractometer
Absorption correction: multi-scan (*CrystalClear*; Rigaku, 2005 ▶) *T*_min_ = 0.884, *T*_max_ = 0.950
6395 measured reflections
2799 independent reflections
1732 reflections with *I* > 2σ(*I*)
*R*_int_ = 0.049

#### Refinement

*R*[*F* ^2^ > 2σ(*F* ^2^)] = 0.058
*wR*(*F* ^2^) = 0.237
*S* = 0.99
2799 reflections
183 parameters
H-atom parameters constrained
Δρ_max_ = 0.58 e Å^−3^
Δρ_min_ = −0.53 e Å^−3^

### 

Data collection: *CrystalClear* (Rigaku 2005 ▶); cell refinement: *CrystalClear*; data reduction: *CrystalClear*; program(s) used to solve structure: *SHELXS97* (Sheldrick, 2008 ▶); program(s) used to refine structure: *SHELXL97* (Sheldrick, 2008 ▶); molecular graphics: *SHELXTL* (Sheldrick, 2008 ▶); software used to prepare material for publication: *PRPKAPPA* (Ferguson, 1999 ▶).

## Supplementary Material

Crystal structure: contains datablocks I, global. DOI: 10.1107/S1600536809051319/rz2399sup1.cif

Structure factors: contains datablocks I. DOI: 10.1107/S1600536809051319/rz2399Isup2.hkl

Additional supplementary materials:  crystallographic information; 3D view; checkCIF report

## Figures and Tables

**Table 1:** Hydrogen-bond geometry (Å, °)

*D*—H⋯*A*	*D*—H	H⋯*A*	*D*⋯*A*	*D*—H⋯*A*
N2—H2*A*⋯O4	0.89	2.36	2.953 (4)	124
N2—H2*A*⋯O8	0.89	2.15	2.884 (5)	139
N2—H2*B*⋯O5^i^	0.89	2.31	3.113 (5)	150
N2—H2*B*⋯O6^i^	0.89	2.34	3.120 (5)	147
N2—H2*C*⋯O2^ii^	0.89	2.15	2.960 (5)	152
O3—H3⋯O4^iii^	0.82	1.89	2.690 (4)	167
C2—H2⋯O8^iv^	0.93	2.58	3.420 (5)	150
C6—H6⋯O2^ii^	0.93	2.54	3.445 (5)	163
C8—H8*A*⋯O6^iv^	0.97	2.50	3.403 (5)	154
C8—H8*B*⋯O7^v^	0.97	2.57	3.134 (6)	117

Symmetry codes: (i) 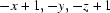; (ii) 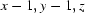; (iii) 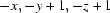; (iv) 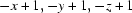; (v) 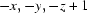.

**Fig. 1.**
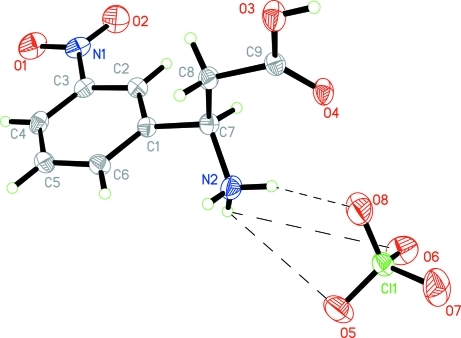
The asymmetric unit in the title compound, with the displacement ellipsoids were drawn at the 30% probability level. Intramolecular hydrogen bonds are shown as dashed lines.

## supplementary crystallographic information

### Comment

β-Amino acids are important molecules due to their pharmacological properties. Recently, there have been an increased interest in the enantiomeric preparation of β-amino acids as precursors for the synthesis of novel biologically active compounds (Cohen *et al.*, 2002). In addition, β-amino acids are attractive ligands for use in the generation of polar coordination polymers, especially when one considers that the ferroelectric compounds (Qu *et al.*, 2004).

The asymmetric unit of the title compound (Fig. 1) contains one 1-(3-nitrophenyl)-2-carboxyethanaminium and one perchlorate anion. The conformation of the cation is stabilized by an intramolecular N—H···O hydrogen bond (Table 1). In the crystal packing, centrosymmetrically related cations at (x, y, z) and (-x, 1-y, 1-z) are linked into a dimer by intermolecular O—H···O hydrogen bonds involving the carboxy groups forming an eigth-membered ring of graph set motif *R*^2^_2_(8) (Etter *et al.*, 1990; Bernstein *et al.*, 1995). The dimers and the perchlorate anions are further connected into layers parallel to the *ab* plane (Fig. 2) by C—H···O and N—H···O hydrogen bonding interactions.

### Experimental

Under nitrogen protection, 3-nitrobenzaldehyde (4.53 g, 30 mmol), malonic acid (5.0 g, 48 mmol) and ammonium acetate (6.0 g, 78 mmol) were added in a flask and refluxed for 12 h yielding a white precipitate. After being cooled to room temperature, the solution was filtered, and the 3-amino-3-(3-nitrophenyl)propanoic acid obtained was dissolved in ethanol and perchloric acid. After slow evaporation of the solution over a period of 3 d, colourless prismatic crystals of the title compound suitable for X-ray diffraction analysis were isolated.

### Refinement

All H atoms were calculated geometrically and were allowed to ride on their parent atoms, with C—H = 0.93–0.97 Å, N—H = 0.89 Å, O—H = 0.82 Å, and with *U*_iso_(H) = 1.2 *U*_eq_(C) or 1.5 *U*_eq_(N, O).

### Crystal data

**Table:**

C_9_H_11_N_2_O_4_^+^·ClO_4_^−^	*Z* = 2
*M**_r_* = 310.65	*F*(000) = 320
Triclinic, *P*1	*D*_x_ = 1.672 Mg m^−^^3^
Hall symbol: -P 1	Mo *K*α radiation, λ = 0.71073 Å
*a* = 7.5932 (8) Å	Cell parameters from 1641 reflections
*b* = 7.8843 (1) Å	θ = 3.1–27.5°
*c* = 11.8615 (6) Å	µ = 0.35 mm^−^^1^
α = 94.745 (3)°	*T* = 293 K
β = 99.780 (7)°	Prism, colourless
γ = 116.323 (4)°	0.45 × 0.30 × 0.15 mm
*V* = 617.11 (7) Å^3^	

### Data collection

**Table:**

Rigaku SCXmini diffractometer	2799 independent reflections
Radiation source: fine-focus sealed tube	1732 reflections with *I* > 2σ(*I*)
graphite	*R*_int_ = 0.049
Detector resolution: 13.6612 pixels mm^-1^	θ_max_ = 27.5°, θ_min_ = 2.9°
CCD Profile fitting scans	*h* = −9→9
Absorption correction: multi-scan (*CrystalClear*; Rigaku, 2005)	*k* = −10→10
*T*_min_ = 0.884, *T*_max_ = 0.950	*l* = −15→15
6395 measured reflections	

### Refinement

**Table:**

Refinement on *F*^2^	Secondary atom site location: difference Fourier map
Least-squares matrix: full	Hydrogen site location: inferred from neighbouring sites
*R*[*F*^2^ > 2σ(*F*^2^)] = 0.058	H-atom parameters constrained
*wR*(*F*^2^) = 0.237	*w* = 1/[σ^2^(*F*_o_^2^) + (0.1426*P*)^2^] where *P* = (*F*_o_^2^ + 2*F*_c_^2^)/3
*S* = 0.99	(Δ/σ)_max_ < 0.001
2799 reflections	Δρ_max_ = 0.58 e Å^−^^3^
183 parameters	Δρ_min_ = −0.53 e Å^−^^3^
0 restraints	Extinction correction: *SHELXL*
Primary atom site location: structure-invariant direct methods	Extinction coefficient: 0.0014 (1)

### Special details

**Table:**

Geometry. All e.s.d.'s (except the e.s.d. in the dihedral angle between two l.s. planes) are estimated using the full covariance matrix. The cell e.s.d.'s are taken into account individually in the estimation of e.s.d.'s in distances, angles and torsion angles; correlations between e.s.d.'s in cell parameters are only used when they are defined by crystal symmetry. An approximate (isotropic) treatment of cell e.s.d.'s is used for estimating e.s.d.'s involving l.s. planes.
Refinement. Refinement of *F*^2^ against ALL reflections. The weighted *R*-factor *wR* and goodness of fit *S* are based on *F*^2^, conventional *R*-factors *R* are based on *F*, with *F* set to zero for negative *F*^2^. The threshold expression of *F*^2^ > σ(*F*^2^) is used only for calculating *R*-factors(gt) *etc*. and is not relevant to the choice of reflections for refinement. *R*-factors based on *F*^2^ are statistically about twice as large as those based on *F*, and *R*- factors based on ALL data will be even larger.

### Fractional atomic coordinates and isotropic or equivalent isotropic displacement parameters (Å^2^)

**Table:**

	*x*	*y*	*z*	*U*_iso_*/*U*_eq_	
Cl1	0.34320 (15)	0.01534 (15)	0.65117 (8)	0.0463 (4)	
O5	0.3304 (6)	−0.1381 (5)	0.5737 (3)	0.0772 (11)	
O6	0.5400 (5)	0.1058 (5)	0.7279 (3)	0.0763 (11)	
O7	0.1979 (6)	−0.0555 (6)	0.7205 (3)	0.0867 (13)	
O8	0.3233 (9)	0.1574 (7)	0.5920 (3)	0.0996 (16)	
O4	0.1696 (4)	0.4174 (4)	0.4775 (2)	0.0423 (7)	
O3	0.0141 (5)	0.5359 (5)	0.3563 (2)	0.0523 (8)	
H3	−0.0234	0.5592	0.4138	0.079*	
C3	0.7013 (5)	0.4845 (5)	0.1024 (3)	0.0343 (8)	
C2	0.6238 (5)	0.4860 (5)	0.1994 (3)	0.0346 (8)	
H2	0.6888	0.5908	0.2602	0.042*	
C1	0.4454 (5)	0.3264 (5)	0.2039 (3)	0.0315 (7)	
N1	0.8866 (5)	0.6592 (5)	0.0985 (3)	0.0467 (9)	
N2	0.2618 (5)	0.1458 (5)	0.3442 (3)	0.0444 (8)	
H2A	0.2265	0.1591	0.4110	0.067*	
H2B	0.3490	0.0984	0.3536	0.067*	
H2C	0.1525	0.0659	0.2896	0.067*	
C9	0.1278 (5)	0.4556 (5)	0.3827 (3)	0.0345 (8)	
C7	0.3587 (5)	0.3383 (5)	0.3081 (3)	0.0338 (8)	
H7	0.4696	0.4278	0.3728	0.041*	
C6	0.3520 (5)	0.1742 (5)	0.1112 (3)	0.0366 (8)	
H6	0.2322	0.0683	0.1136	0.044*	
O1	0.9505 (5)	0.6627 (5)	0.0093 (3)	0.0696 (10)	
C5	0.4334 (6)	0.1763 (6)	0.0143 (3)	0.0386 (8)	
H5	0.3682	0.0730	−0.0475	0.046*	
O2	0.9618 (5)	0.7907 (5)	0.1799 (3)	0.0722 (10)	
C4	0.6139 (6)	0.3347 (6)	0.0106 (3)	0.0394 (9)	
H4	0.6728	0.3382	−0.0524	0.047*	
C8	0.2089 (6)	0.4171 (6)	0.2816 (3)	0.0370 (8)	
H8A	0.2741	0.5361	0.2528	0.044*	
H8B	0.0960	0.3263	0.2198	0.044*	

### Atomic displacement parameters (Å^2^)

**Table:**

	*U*^11^	*U*^22^	*U*^33^	*U*^12^	*U*^13^	*U*^23^
Cl1	0.0496 (6)	0.0469 (6)	0.0457 (6)	0.0250 (5)	0.0138 (4)	0.0040 (4)
O5	0.077 (3)	0.055 (2)	0.092 (3)	0.0231 (19)	0.034 (2)	−0.0077 (19)
O6	0.054 (2)	0.071 (2)	0.082 (2)	0.0171 (19)	−0.0018 (18)	0.013 (2)
O7	0.062 (2)	0.103 (3)	0.084 (3)	0.023 (2)	0.040 (2)	0.002 (2)
O8	0.185 (5)	0.119 (3)	0.047 (2)	0.119 (4)	0.017 (2)	0.022 (2)
O4	0.0490 (15)	0.0624 (18)	0.0341 (14)	0.0396 (15)	0.0155 (12)	0.0111 (12)
O3	0.0618 (18)	0.086 (2)	0.0394 (14)	0.0580 (18)	0.0179 (14)	0.0155 (15)
C3	0.0284 (17)	0.041 (2)	0.0417 (19)	0.0200 (16)	0.0139 (15)	0.0149 (16)
C2	0.0292 (18)	0.0376 (19)	0.0381 (19)	0.0168 (16)	0.0071 (15)	0.0057 (15)
C1	0.0296 (17)	0.0360 (18)	0.0319 (17)	0.0174 (15)	0.0082 (14)	0.0056 (14)
N1	0.0353 (18)	0.049 (2)	0.065 (2)	0.0217 (17)	0.0210 (17)	0.0245 (19)
N2	0.056 (2)	0.052 (2)	0.0401 (17)	0.0335 (18)	0.0215 (16)	0.0162 (15)
C9	0.0336 (18)	0.0391 (19)	0.0350 (18)	0.0198 (16)	0.0111 (15)	0.0045 (15)
C7	0.0297 (17)	0.0371 (19)	0.0349 (18)	0.0149 (15)	0.0101 (14)	0.0070 (15)
C6	0.0341 (18)	0.0330 (18)	0.0413 (19)	0.0137 (16)	0.0119 (15)	0.0046 (15)
O1	0.062 (2)	0.080 (2)	0.088 (2)	0.0352 (19)	0.053 (2)	0.041 (2)
C5	0.042 (2)	0.040 (2)	0.0376 (19)	0.0215 (18)	0.0115 (16)	0.0028 (16)
O2	0.055 (2)	0.054 (2)	0.074 (2)	−0.0025 (17)	0.0133 (18)	0.0074 (19)
C4	0.043 (2)	0.056 (2)	0.0359 (18)	0.032 (2)	0.0193 (16)	0.0173 (17)
C8	0.043 (2)	0.045 (2)	0.0356 (18)	0.0273 (18)	0.0198 (16)	0.0116 (16)

### Geometric parameters (Å, °)

**Table:**

Cl1—O5	1.414 (3)	N1—O1	1.234 (4)
Cl1—O8	1.419 (4)	N2—C7	1.501 (5)
Cl1—O7	1.429 (4)	N2—H2A	0.8900
Cl1—O6	1.433 (4)	N2—H2B	0.8900
O4—C9	1.214 (4)	N2—H2C	0.8900
O3—C9	1.292 (4)	C9—C8	1.506 (4)
O3—H3	0.8200	C7—C8	1.521 (5)
C3—C4	1.366 (5)	C7—H7	0.9800
C3—C2	1.380 (5)	C6—C5	1.392 (5)
C3—N1	1.481 (5)	C6—H6	0.9300
C2—C1	1.395 (5)	C5—C4	1.397 (5)
C2—H2	0.9300	C5—H5	0.9300
C1—C6	1.384 (5)	C4—H4	0.9300
C1—C7	1.511 (5)	C8—H8A	0.9700
N1—O2	1.207 (5)	C8—H8B	0.9700
			
O5—Cl1—O8	111.9 (2)	O4—C9—O3	125.3 (3)
O5—Cl1—O7	110.5 (2)	O4—C9—C8	122.8 (3)
O8—Cl1—O7	111.6 (3)	O3—C9—C8	111.9 (3)
O5—Cl1—O6	107.7 (2)	N2—C7—C1	111.0 (3)
O8—Cl1—O6	107.2 (3)	N2—C7—C8	111.1 (3)
O7—Cl1—O6	107.7 (2)	C1—C7—C8	110.1 (3)
C9—O3—H3	109.5	N2—C7—H7	108.2
C4—C3—C2	123.7 (3)	C1—C7—H7	108.2
C4—C3—N1	119.4 (3)	C8—C7—H7	108.2
C2—C3—N1	116.9 (3)	C1—C6—C5	121.4 (3)
C3—C2—C1	118.3 (3)	C1—C6—H6	119.3
C3—C2—H2	120.9	C5—C6—H6	119.3
C1—C2—H2	120.9	C6—C5—C4	119.4 (3)
C6—C1—C2	119.1 (3)	C6—C5—H5	120.3
C6—C1—C7	124.0 (3)	C4—C5—H5	120.3
C2—C1—C7	116.7 (3)	C3—C4—C5	117.9 (3)
O2—N1—O1	124.1 (4)	C3—C4—H4	121.0
O2—N1—C3	118.7 (3)	C5—C4—H4	121.0
O1—N1—C3	117.1 (4)	C9—C8—C7	115.3 (3)
C7—N2—H2A	109.5	C9—C8—H8A	108.4
C7—N2—H2B	109.5	C7—C8—H8A	108.4
H2A—N2—H2B	109.5	C9—C8—H8B	108.4
C7—N2—H2C	109.5	C7—C8—H8B	108.4
H2A—N2—H2C	109.5	H8A—C8—H8B	107.5
H2B—N2—H2C	109.5		

### Hydrogen-bond geometry (Å, °)

**Table:**

*D*—H···*A*	*D*—H	H···*A*	*D*···*A*	*D*—H···*A*
N2—H2A···O4	0.89	2.36	2.953 (4)	124
N2—H2A···O8	0.89	2.15	2.884 (5)	139
N2—H2B···O5^i^	0.89	2.31	3.113 (5)	150
N2—H2B···O6^i^	0.89	2.34	3.120 (5)	147
N2—H2C···O2^ii^	0.89	2.15	2.960 (5)	152
O3—H3···O4^iii^	0.82	1.89	2.690 (4)	167
C2—H2···O8^iv^	0.93	2.58	3.420 (5)	150
C6—H6···O2^ii^	0.93	2.54	3.445 (5)	163
C8—H8A···O6^iv^	0.97	2.50	3.403 (5)	154
C8—H8B···O7^v^	0.97	2.57	3.134 (6)	117

Symmetry codes: (i) −*x*+1, −*y*, −*z*+1; (ii) *x*−1, *y*−1, *z*; (iii) −*x*, −*y*+1, −*z*+1; (iv) −*x*+1, −*y*+1, −*z*+1; (v) −*x*, −*y*, −*z*+1.
